# The Effect of a Temporary Stoma on Long-term Functional Outcomes Following Surgery for Rectal Cancer

**DOI:** 10.1097/DCR.0000000000003009

**Published:** 2023-12-20

**Authors:** Sanne J. Verkuijl, Jara E. Jonker, Edgar J.B. Furnée, Wendy Kelder, Christiaan Hoff, Daniel A. Hess, Fennie Wit, Ronald J. Zijlstra, Monika Trzpis, Paul M.A. Broens

**Affiliations:** 1 Department of Surgery, Anorectal Physiology Laboratory, University of Groningen, University Medical Center Groningen, Groningen, The Netherlands; 2 Division of Abdominal Surgery, Department of Surgery, University of Groningen, University Medical Center Groningen, Groningen, The Netherlands; 3 Department of Surgery, Martini Hospital, Groningen, The Netherlands; 4 Department of Surgery, Medical Centre Leeuwarden, Leeuwarden, The Netherlands; 5 Department of Surgery, Antonius Hospital, Sneek, The Netherlands; 6 Department of Surgery, Tjongerschans Hospital, Heerenveen, The Netherlands; 7 Department of Surgery, Nij Smellinghe Hospital, Drachten, The Netherlands; 8 Division of Pediatric Surgery, Department of Surgery, University of Groningen, University Medical Center Groningen, Groningen, The Netherlands

**Keywords:** Bowel dysfunction, Follow-up, Postoperative, Rectal cancer, Stoma

## Abstract

**BACKGROUND:**

Patients with rectal cancer may undergo surgical resection with or without a temporary stoma.

**OBJECTIVE:**

This study primarily aimed to compare long-term functional outcomes between patients with and without a temporary stoma after surgery for rectal cancer. The secondary aim was to investigate the effect of time to stoma reversal on functional outcomes.

**DESIGN:**

This was a multicenter, cross-sectional study.

**SETTINGS:**

This study was conducted at 7 Dutch hospitals.

**PATIENTS:**

Included were patients who had undergone rectal cancer surgery (2009–2015). Excluded were deceased patients, who were deceased, had a permanent stoma, or had intellectual disability.

**MAIN OUTCOME MEASURES:**

Functional outcomes were measured using the Rome IV criteria for constipation and fecal incontinence and the low anterior resection syndrome score.

**RESULTS:**

Of 656 patients, 32% received a temporary ileostomy and 20% a temporary colostomy (86% response). Follow-up was at 56 (interquartile range, 38.5–79) months. Patients who had a temporary ileostomy experienced less constipation, more fecal incontinence, and more major low anterior resection syndrome than those without a temporary stoma. Patients who had a temporary colostomy experienced more major low anterior resection syndrome than those without a temporary stoma. A temporary ileostomy or colostomy was not associated with constipation or fecal incontinence after correction for confounding factors (eg, anastomotic height, anastomotic leakage, radiotherapy). Time to stoma reversal was not associated with constipation, fecal incontinence, or major low anterior resection syndrome.

**LIMITATIONS:**

Cross-sectional design.

**CONCLUSIONS:**

Although patients with a temporary ileostomy or colostomy have worse functional outcomes in the long term, it seems that the reason for creating a temporary stoma, rather than the stoma itself, underlies this phenomenon. Time to reversal of a temporary stoma does not influence functional outcomes. See **Video Abstract**.

**EL EFECTO DEL ESTOMA TEMPORAL SOBRE LOS RESULTADOS FUNCIONALES A LARGO PLAZO DESPUÉS DE LA CIRUGÍA POR CÁNCER DE RECTO:**

**ANTECEDENTES:**

Los pacientes con cáncer de recto pueden someterse a resección quirúrgica con o sin un estoma temporal.

**OBJETIVO:**

El objetivo principal de este estudio fue comparar los resultados funcionales a largo plazo entre pacientes con y sin estoma temporal después de cirugía por cáncer de recto. El objetivo secundario fue investigar el efecto del tiempo transcurrido hasta la reversión del estoma sobre los resultados funcionales.

**DISEÑO:**

Este fue un estudio transversal multicéntrico.

**ESCENARIO:**

Este estudio se llevó a cabo en siete hospitales holandeses.

**PACIENTES:**

Se incluyeron pacientes sometidos a cirugía de cáncer de recto (2009-2015). Se excluyeron pacientes fallecidos, pacientes con estoma permanente o discapacidad intelectual.

**PRINCIPALES MEDIDAS DE RESULTADO:**

Los resultados funcionales se midieron utilizando los criterios de Roma IV para el estreñimiento y la incontinencia fecal y la puntuación del síndrome de resección anterior baja (LARS).

**RESULTADOS:**

De 656 pacientes, el 32% recibió una ileostomía temporal y el 20% una colostomía temporal (respuesta del 86%). El seguimiento fue de 56.0 (RIQ 38.5-79.0) meses. Los pacientes a los que se les realizó una ileostomía temporal experimentaron menos estreñimiento, más incontinencia fecal y más LARS mayor que los pacientes sin un estoma temporal. Los pacientes que tuvieron una colostomía temporal experimentaron más LARS mayor que los pacientes sin un estoma temporal. Una ileostomía o colostomía temporal no se asoció con estreñimiento o incontinencia fecal después de la corrección de factores de confusión (p. ej., altura anastomótica, fuga anastomótica, radioterapia). El tiempo hasta la reversión del estoma no se asoció con estreñimiento, incontinencia fecal o LARS mayor.

**LIMITACIONES:**

El presente estudio está limitado por su diseño transversal.

**CONCLUSIONES:**

Aunque los pacientes con una ileostomía o colostomía temporal tienen peores resultados funcionales a largo plazo, parece que la razón para crear un estoma temporal, más que el estoma en sí, se asocia a este fenómeno. El tiempo hasta la reversión de un estoma temporal no influye en los resultados funcionales. *(Traducción—Dr. Jorge Silva Velazco*)

More than 700,000 people worldwide are affected by rectal cancer every year. Although the incidence of rectal cancer has increased over the past decades, its mortality has decreased.^1,2^ The rise in incidence is generally attributed to obesity, lifestyle, and dietary changes, whereas the decrease in mortality seems to be caused by improvements in early detection, chemotherapy and radiotherapy, perioperative care, and surgical techniques.^3–5^

Low anterior resection is still considered the standard for the curative treatment of proximal rectum or distal sigmoid tumors provided an adequate distal resection margin can be achieved.^6,7^ Following low anterior resection, bowel continuity can be directly restored by creating an anastomosis. The surgeon may, however, also opt for a temporary stoma. Creating a temporary stoma is thought to prevent high morbidity and mortality, especially if the patient underwent neoadjuvant radiotherapy, has a low anastomosis, or when anastomotic leakage occurs.^8,9^ Nevertheless, there is no consensus on long-term functional outcomes and quality of life after stoma reversal.^10–13^ Furthermore, the best timing of stoma reversal in relation to postoperative functional outcomes is under debate.^14–16^ This impairs preoperative patient counseling regarding the creation of a temporary stoma, as well as evidence-based decision-making about the best time to reverse a stoma.

We hypothesized that long-term functional outcomes after stoma reversal are worse compared to patients without a temporary stoma because of reasons to create a stoma (such as a lower anastomosis or neoadjuvant radiotherapy) as well as factors such as temporary afunctional anorectal muscles and differences in colonic microflora.

Our primary aim was to investigate the difference in long-term functional outcomes and quality of life between patients with and without a temporary stoma after surgery for rectal cancer. Our secondary aim was to investigate the effect of time-to-stoma reversal on long-term functional outcomes and quality of life.

## MATERIALS AND METHODS

This cross-sectional study was performed in 7 hospitals in the north of The Netherlands between October 2017 and December 2019. The clinical records of all patients without a permanent stoma who had undergone low anterior resection or anterior resection because of rectal or rectosigmoid cancer between 2009 and 2015 were reviewed. Low anterior resection or anterior resection was defined as a total mesorectal excision with an anastomosis <15 cm from the anal verge.^6,7^ Anastomotic leakage was noted if confirmed by endoscopy, radiology, or surgery. Patients who underwent neoadjuvant radiotherapy were given a total dose of 25 Gy in 5 fractions (short course) or 45 to 50.4 Gy in 25 to 28 fractions (long course). Long-course neoadjuvant radiotherapy was usually combined with chemotherapy.

Patients were identified through the mandatory Dutch ColoRectal Audit registry. We excluded deceased patients, patients with intellectual disability, patients younger than 18 years at the time of surgery, and patients who had emigrated or whose correspondence address was unknown to us. After the eligible patients had given us their written informed consent, they were sent the validated Defecation and Fecal Continence (DeFeC) and the Short Form 36 questionnaires.^17–19^ The questionnaires could be completed on paper or online, depending on the individual patient’s preference.

This study was conducted after approval by the Medical Ethical Review Board of the University Medical Center Groningen, The Netherlands (approval code: METc 2017/245).

### Primary and Secondary Outcome Measures

The primary outcomes were constipation, fecal incontinence, and low anterior resection syndrome (LARS) score. Constipation and fecal incontinence were defined according to the Rome IV criteria,^20,21^ which are included in the DeFeC questionnaire. Constipation was defined as having at least 2 of the following symptoms: straining, hard or lumpy stool, sense of obstruction, incomplete defection, manual facilitation of defecation, and/or less than 3 stools per week.^20^ Fecal incontinence was defined as having recurrent, uncontrolled passage of stool at least several times per month.^21^ The severity of constipation was measured using the Agachan score and the severity of fecal incontinence was measured using the Wexner score, which are also implemented in the DeFeC questionnaire.^22,23^ The Agachan score ranges from 0 to 30, where 0 indicates no constipation and 30 indicates severe constipation.^22^ The Wexner score ranges from 0 to 20, where 0 indicates perfect fecal continence and 20 indicates complete fecal incontinence.^23^ The LARS score is also implemented in the DeFeC questionnaire. It consists of 5 questions on incontinence for flatus or liquid stools, stool frequency, fecal clustering, and urgency.^24^ The LARS score ranges from 0 to 42, where 0 to 20 indicates no LARS, 21 to 29 indicates minor LARS, and 30 or greater indicates major LARS, as originally defined by Emmertsen and Laurberg.^24^

The secondary outcome measure was quality of life according to the Short Form 36 questionnaire. Eight quality-of-life domains can be calculated from this questionnaire, each with a scale ranging from 0 to 100, where a higher score indicates a better quality of life.^18^

### Statistical Analyses

Data were analyzed using SPSS version 23.0 for Windows (SPSS Statistics, IBM Corporation, Armonk, NY). Given their skewed distribution, values were presented as numbers (percentages) or medians (interquartile ranges [IQR]). Comparisons between the groups of patients without a previous stoma, with a previous ileostomy, or with a previous colostomy were performed using the Pearson χ^2^ test for categorical variables and the Kruskal-Wallis test for continuous variables. The Mann-Whitney *U* test was applied for 2-by-2 subgroup comparisons with the Bonferroni post hoc correction to decrease the risk of a type I error. Univariable and multivariable logistic regressions were performed to investigate the association between the presence of a temporary stoma and constipation, fecal incontinence, and major LARS. The associations were reported as ORs with 95% CIs. Variables with a *p* value of <0.10 in univariable regression analysis or variables with a proven theoretical confounding effect according to the literature were included as confounding variables in multivariable regression analysis. The Spearman rank correlation test was applied to test for correlations between the length of time with a stoma and bowel function outcomes. A correlation coefficient <0.3 was considered negligible. Figures were created using GraphPad Prism version 8.4.2 for Windows (GraphPad Software Inc., La Jolla, CA). R version 3.6.3 (R Foundation of Statistical Computing, Vienna, Austria) was used for the cubic spline regression graphs. The level of statistical significance was preset at a *p* value of <0.05.

## RESULTS

We identified 1071 patients who underwent surgery for rectal cancer. After excluding 312 patients, we sent questionnaires to 759 eligible patients. In total, we received completed questionnaires from 656 patients, which is a response rate of 86%. The flow chart of the study population and a comparison of the responders versus the nonresponders were described previously.^25^ Age was the only variable on which the responders differed significantly from the nonresponders (65.0 versus 67.0, *p* = 0.025). The number of patients with an ileostomy or colostomy did not differ between the responders and the nonresponders (*p* = 0.932; see Supplemental Digital Content 1 at http://links.lww.com/DCR/C243).

### Patient Characteristics

The majority of the study population was male (62%). The patients’ median age at surgery was 65.0 (IQR, 58.0–69.0) years and the median time to follow-up was 56.0 (IQR, 38.5–79.0) months. A temporary ileostomy was created in 208 (32%) patients and 130 (20%) patients had a temporary colostomy. Most patients received their temporary ileostomy (95%) or colostomy at primary surgery (73%). Table 1 lists the clinical characteristics of the included patients according to whether they received no stoma, a previous ileostomy, or a previous colostomy. There were significantly more male patients in the ileostomy subgroup compared to the no stoma subgroup (71% versus 56%, *p* < 0.001). The ASA score, tumor stage, and presence of distant metastases did not differ significantly between the subgroups without a stoma, with a previous ileostomy, or with a previous colostomy. There were significantly fewer patients who received neoadjuvant radiotherapy in the no stoma subgroup compared to the subgroups with a previous ileostomy or colostomy (22% versus 80%, *p* < 0.001 and 22% versus 68%, *p* < 0.001, respectively). The total dose of radiotherapy was significantly lower in patients with a previous ileostomy compared to patients with a previous colostomy (short course in 60% versus 44%, *p* = 0.011). Significantly more patients without a stoma received adjuvant chemotherapy compared to the patients with a previous ileostomy or colostomy (25% versus 12%, *p* < 0.001 and 25% versus 14%, *p* = 0.008, respectively). In Supplemental Digital Content 2 (at http://links.lww.com/DCR/C244), we report the underlying reasons for constructing a temporary ileostomy or colostomy.

**TABLE 1.** Patient characteristics according to the previous presence of a temporary stoma

*Patient characteristics*	*No stoma, n (%*)	*Ileostomy, n (%*)	*Colostomy, n (%*)	*p* a	*p* b
Overall	318 (100)	208 (100)	130 (100)		
Timing of stoma creation At primary surgery Prior to primary surgery After primary surgery		197 (94.7) 2 (1.0) 9 (4.3)	95 (73.1) 28 (21.5) 7 (5.4)	–	<0.001**
Male patients	177 (55.7)	148 (71.2)	82 (63.1)	0.002**	0.121
Age at surgery,^c^ y	65.0 (60.0–70.0)	64.0 (56.0–68.0)	63.0 (57.0–69.0)	0.013*	0.996
Follow-up,^c,d^ mo	60.0 (40.0–82.0)	57.0 (36.5–78.0)	48.0 (38.0–75.0)	0.056	0.542
ASA score at surgery I II III IV	95 (31.1) 169 (55.4) 39 (12.8) 2 (0.7)	69 (33.3) 123 (59.4) 15 (7.2) 0 (0.0)	37 (28.9) 75 (58.6) 15 (11.7) 1 (0.8)	0.423	0.274
Tumor stage (UICC) I II III IV	111 (35.0) 96 (30.3) 100 (31.5) 10 (3.2)	81 (38.9) 71 (34.1) 53 (25.5) 3 (1.4)	48 (36.9) 40 (30.8) 36 (27.7) 6 (4.6)	0.452	0.316
Distant metastasis^e^ No Liver Lung Multiple locations	293 (92.1) 15 (4.7) 5 (1.6) 5 (1.6)	189 (90.9) 11 (5.3) 5 (2.4) 3 (1.4)	112 (86.2) 11 (8.5) 6 (4.6) 1 (0.8)	0.352	0.400
Neoadjuvant radiotherapy	68 (21.9)	164 (79.6)	85 (67.5)	<0.001**	0.013*
Total dose of radiotherapy Short course (25 Gy) Long course (45–50.4 Gy)	57 (83.8) 11 (16.2)	99 (60.4) 65 (39.6)	37 (43.5) 48 (56.5)	<0.001**	0.011*
Time since last radiotherapy,^c^ y	6.0 (5.0-8.0)	6.0 (4.0-7.0)	6.0 (4.0-8.0)	0.075	0.775
Adjuvant chemotherapy	79 (25.4)	25 (12.1)	18 (14.0)	<0.001**	0.617
Emergency setting	14 (4.6)	1 (0.5)	8 (6.2)	0.010*	0.002**
Surgical approach Open Laparoscopic Conversion	87 (27.4) 209 (65.7) 22 (6.9)	64 (30.9) 119 (57.5) 24 (11.6)	80 (61.5) 43 (33.1) 7 (5.4)	<0.001**	<0.001**
Anastomotic height,^c^ cm	10.0 (8.0–15.0)	6.0 (5.0–9.0)	5.0 (4.0–9.0)	<0.001**	0.069
Method of primary anastomosis Hand-sewn Stapled	26 (8.3) 288 (91.7)	3 (1.5) 199 (98.5)	3 (2.3) 126 (97.7)	0.001**	0.567
Reconstruction of primary anastomosis Side-to-end Side-to-side End-to-end	208 (74.6) 11 (3.9) 60 (21.5)	130 (78.8) 3 (1.8) 32 (19.4)	98 (85.2) 5 (4.3) 12 (10.4)	0.078	0.070
Anastomotic leakage	5 (1.6)^f^	19 (9.1)^g^	15 (11.5)^h^	<0.001**	0.475

IQR = interquartile range; UICC = Union for International Cancer Control.

a Comparison between the 3 subgroups (no stoma versus ileostomy versus colostomy).

b Comparison between the following subgroups: ileostomy versus colostomy.

c Values are expressed as median (IQR).

d Time between primary surgery or stoma reversal (no stoma subgroup) and completing the questionnaires.

e At the time of completing the questionnaires.

f Patients with anastomotic leakage treated conservatively.

g Patients with anastomotic leakage treated with a temporary stoma (n = 9) or patients who received a temporary stoma at primary surgery, but with clear abscess formation around the primary anastomosis postoperatively (n = 10).

h Patients with anastomotic leakage treated with a temporary stoma (n = 7) or patients who received a temporary stoma before or at primary surgery, but with clear abscess formation around the primary anastomosis postoperatively (n = 8).

* Statistical significance of *p* < 0.05.

** Statistical significance of *p* < 0.005.

### Functional Outcomes With and Without a Temporary Stoma

**FIGURE 1.**
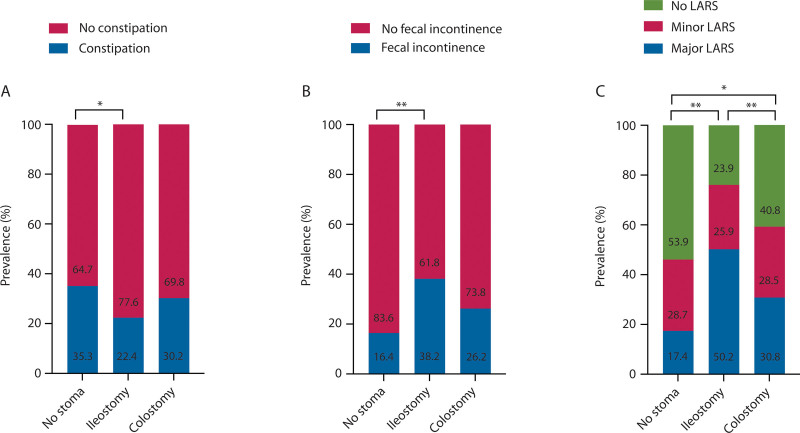
The prevalence of constipation (A), fecal incontinence (B), and major LARS (C) and a previous temporary stoma. LARS = low anterior resection syndrome. *Statistical significance preset at *p* < 0.05. **Statistical significance preset at *p* < 0.005.

**FIGURE 2.**
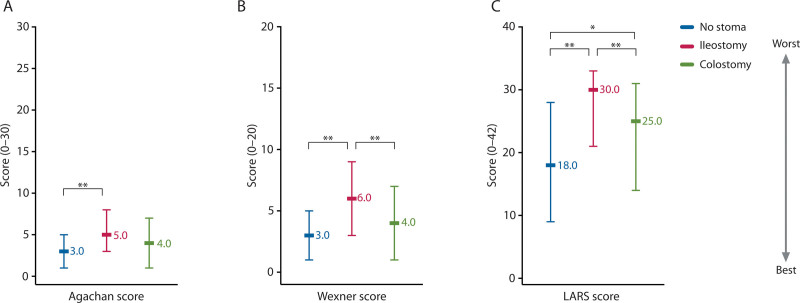
The severity of constipation (A), fecal incontinence (B), and LARS (C) and a previous temporary stoma. LARS = low anterior resection syndrome. *Statistical significance preset at *p* < 0.05. **Statistical significance preset at *p* < 0.005.

The prevalence of constipation was significantly lower in patients with a previous ileostomy compared to patients without a previous stoma (22% versus 35%, *p* = 0.002; Fig. 1A). In contrast, the prevalence of fecal incontinence and major LARS was significantly higher in patients with a previous ileostomy compared to patients without a previous stoma (38% versus 16%, *p* < 0.001; Fig. 1B; and 50% versus 17%, *p* < 0.001; Fig. 1C). The severity score of fecal incontinence and the LARS score were significantly higher in patients with a previous ileostomy compared to patients without a previous stoma (6.0 versus 3.0, *p* < 0.001 and 30.0 versus 18.0, *p* < 0.001; Fig. 2). Univariable logistic regression analysis showed that patients with a previous ileostomy were less likely to experience constipation (OR, 0.53; 95% CI, 0.36–0.79; *p* = 0.002; Table 2), but this was no longer significant following multivariable regression analysis. The likelihood of fecal incontinence was higher in patients with a previous ileostomy according to univariable logistic regression analysis (OR, 3.16; 95% CI, 2.10–4.75; *p* < 0.001; Table 2), but this significant difference also disappeared following multivariable logistic regression analysis. Regarding major LARS, both univariable and multivariable logistic regression analyses revealed a significantly higher likelihood of major LARS in patients with a previous ileostomy (OR, 4.18; 95% CI, 3.23–7.17; *p* < 0.001 and OR 1.94; 95% CI, 1.16–3.25; *p* = 0.012, respectively; Table 2).

**TABLE 2.** Univariable and multivariable logistic regression analyses of constipation, fecal incontinence, and major LARS

*Variables*	*Constipation*				*Fecal incontinence*				*Major LARS*			
	*Univariable*		*Multivariable*		*Univariable*		*Multivariable*		*Univariable*		*Multivariable*	
	*OR (95% CI*)	*p*	*OR (95% CI*)	*p*	*OR (95% CI*)	*p*	*OR (95% CI*)	*p*	*OR (95% CI*)	*p*	*OR (95% CI*)	*p*
Temporary stoma No Ileostomy Colostomy	Reference 0.53 (0.36–0.79) 0.79 (0.51–1.23)	0.002** 0.303	Reference 0.85 (0.50–1.42) 1.16 (0.67–2.00)	0.528 0.599	Reference 3.16 (2.10–4.75) 1.81 (1.11–2.96)	<0.001** 0.018*	Reference 1.18 (0.69–2.01) 0.72 (0.39–1.33)	0.551 0.289	Reference 4.81 (3.23–7.17) 2.12 (1.32–3.40)	<0.001** 0.002**	Reference 1.94 (1.16–3.25) 0.88 (0.49–1.60)	0.012* 0.882
Anastomotic height, cm	1.10 (1.05–1.14)	<0.001**	1.08 (1.02–1.15)	0.013*	0.87 (0.82–0.91)	<0.001**	0.91 (0.85–0.97)	0.005**	0.85 (0.80–0.89)	<0.001**	0.92 (0.86–0.98)	0.010*
Sex Men Women	Reference 1.36 (0.96–1.91)	0.081	Reference 1.27 (0.88–1.84)	0.207	Reference 0.72 (0.49–1.04)	0.079	Reference 0.76 (0.50–1.16)	0.205	Reference 0.80 (0.56–1.13)	0.200	Reference 0.87 (0.58–1.31)	0.503
Age at surgery, y	1.00 (0.98–1.02)	0.814	0.99 (0.97–1.01)	0.264	0.98 (0.96–1.00)	0.043*	0.99 (0.97–1.02)	0.551	0.99 (0.97–1.01)	0.188	1.01 (0.98–1.03)	0.672
Follow-up, mo	1.01 (1.00–1.01)	0.126	1.00 (0.99–1.01)	0.964	1.00 (0.99–1.01)	0.665	1.00 (0.99–1.01)	0.620	1.00 (1.00–1.01)	0.700	1.00 (0.99–1.01)	0.501
ASA score at surgery	1.31 (1.00–1.71)	0.050	1.37 (1.01–1.86)	0.042*	0.80 (0.60–1.07)	0.131	–	–	1.09 (0.83–1.42)	0.546	*–*	–
Radiotherapy No Yes	Reference 0.61 (0.44–0.86)	0.005*	Reference 0.95 (0.58–1.56)	0.853	Reference 3.64 (2.46–5.38)	<0.001**	Reference 2.42 (1.43–4.10)	0.001**	Reference 5.03 (3.44–7.35)	<0.001**	Reference 2.94 (1.77–4.88)	<0.001**
Total dose of radiotherapy Short course Long course	Reference 0.86 (0.51–1.46)	0.577	–	–	Reference 1.19 (0.74–1.90)	0.471	–	–	Reference 0.74 (0.47–1.17)	0.193	*–*	–
Adjuvant chemotherapy No Yes	Reference 1.51 (1.00–2.28)	0.051	Reference 1.25 (0.78–2.02)	0.358	Reference 0.86 (0.54–1.37)	0.520	–	–	Reference 0.54 (0.34–0.87)	0.011*	Reference 0.75 (0.42–1.37)	0.352
Anastomotic leakage No Yes	Reference 1.16 (0.59–2.31)	0.667	–	–	Reference 3.42 (1.78–6.58)	<0.001**	Reference 2.30 (1.10–4.82)	0.027*	Reference 2.06 (1.07–3.96)	0.030*	Reference 1.42 (0.66–3.05)	0.375

LARS = low anterior resection syndrome; UICC = Union for International Cancer Control.

* Statistical significance of *p* < 0.05.

** Statistical significance of *p* < 0.005.

In patients with a previous colostomy, the prevalence of major LARS was significantly higher compared to patients without a previous stoma (31% versus 17%, *p* = 0.002; Fig. 1C). In addition, the LARS score was higher in patients with a previous colostomy compared to patients without a previous stoma (25.0 versus 18.0, *p* = 0.003; Fig. 2C). Univariable logistic regression analysis did not show an association between a previous colostomy and constipation. By contrast, patients with a previous colostomy were more likely to experience fecal incontinence and major LARS following univariable logistic regression analysis (OR, 1.81; 95% CI, 1.11–2.96; *p* = 0.018 and OR, 2.12; 95% CI, 1.32–3.40; *p* = 0.002; Table 2). These significant differences disappeared after multivariable logistic regression analysis.

### Functional Outcomes and Time to Stoma Reversal

**FIGURE 3.**
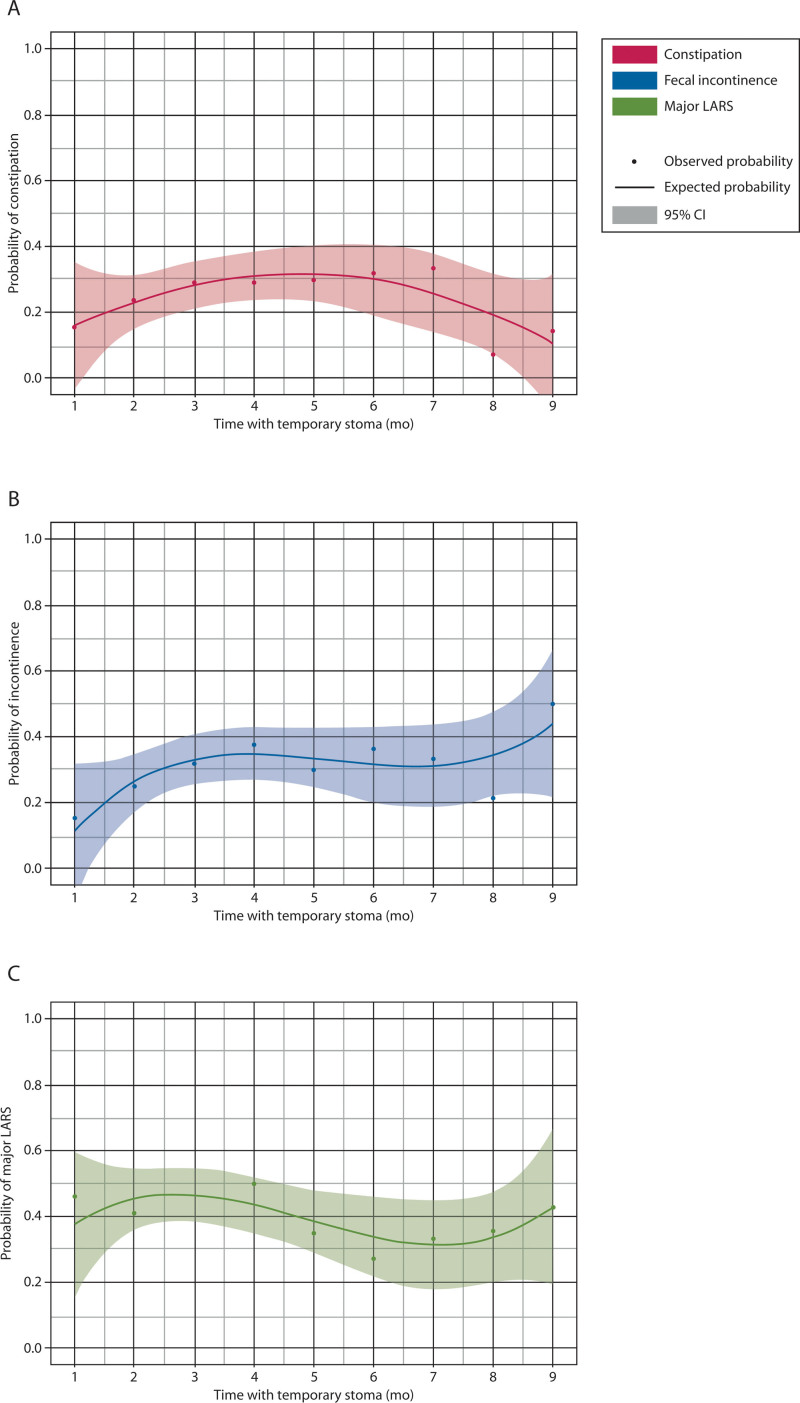
The probability of constipation (A), fecal incontinence (B), and major LARS (C) and time to stoma reversal. This figure only includes data on the included patients who had their stomas reversed within the first 9 mo because this comprised 90% of the total study population. LARS = low anterior resection syndrome.

The median time to stoma reversal was 4.0 (IQR, 3.0–7.0) months, which was significantly shorter in patients with a previous ileostomy compared to patients with a previous colostomy (3.0 versus 5.0, *p* < 0.001). Time to stoma reversal was not significantly associated with constipation (OR, 1.01; 95% CI, 0.97–1.06; *p* = 0.620), fecal incontinence (OR, 1.03; 95% CI, 0.98–1.07; *p* = 0.218), or major LARS (OR, 0.99; 95% CI, 0.95–10.3; *p* = 0.385). In accordance with these results, the probability of constipation, fecal incontinence, and major LARS remained stable between time to stoma reversal from 1 to 9 months after stoma creation (Fig. 3). Likewise, time to stoma reversal was also not significantly correlated with more severe constipation (Agachan score, rho = 0.010, *p* = 0.865), more severe fecal incontinence (Wexner score rho = –0.012, *p* = 0.829), or with a higher LARS score (rho = –0.026, *p* = 0.647).

### Quality of Life With and Without a Temporary Stoma

**FIGURE 4.**
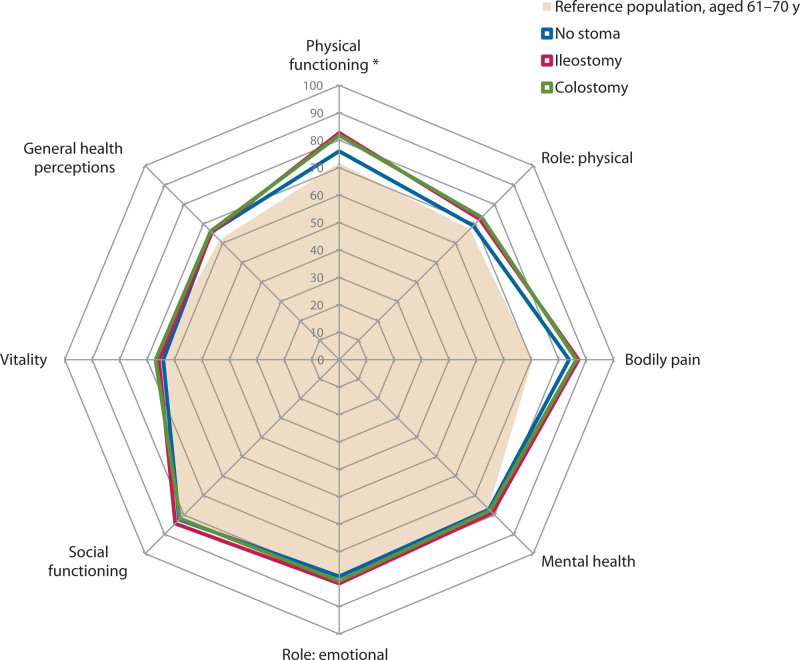
Generic quality-of-life scores and the presence of a temporary stoma.

Patients with a previous ileostomy or colostomy reported better physical functioning compared to patients without a stoma (82.7 versus 76.0, *p* = 0.025, and 81.8 versus 76.0, *p* = 0.042, respectively; Fig. 4). These values did not remain significant after post hoc correction. No other quality-of-life domains were found to be significantly different between the subgroups of patients without a stoma, with a previous ileostomy, or with a previous colostomy.

### Quality of Life and Time to Stoma Reversal

Correlations between time to stoma reversal and the quality-of-life domain scores were only significant for the domains bodily pain and vitality, but both correlation coefficients were negligible (rho = –0.120, *p* = 0.031 and rho = –0.120, *p* = 0.033, respectively; see Supplemental Digital Content 3 at http://links.lww.com/DCR/C245).

## DISCUSSION

To the best of our knowledge, the current study on long-term bowel function and quality of life of patients who underwent surgery for rectal cancer comprises one of the largest study populations. This study showed that patients who received a temporary ileostomy experienced more fecal incontinence and major LARS even years after its reversal. Patients with a temporary colostomy experienced more major LARS. Time to stoma reversal did not seem to affect the long-term functional outcomes or quality of life.

Interestingly, we observed a higher prevalence and higher severity of fecal incontinence and major LARS in patients with a previous ileostomy compared to patients without a temporary stoma. Patients with a previous colostomy showed more major LARS in the long term compared to patients without a temporary stoma. In contrast, it is possible that the construction of the temporary stoma itself results in worse functional outcomes. If the fecal continence mechanisms have not been used for a number of months to years, bowel function problems may occur after stoma reversal and the anorectal muscles may need to be “trained” once again.^26^ This theory is supported by a review showing that pelvic floor rehabilitation after low anterior resection improves bowel function.^27^ In contrast, the underlying reasons for creating the stoma may cause the long-term bowel function problems. This theory is supported by recent meta-analyses.^12,28,29^ The reasons for creating a temporary stoma after rectal cancer surgery vary broadly and may include a low anastomosis, neoadjuvant radiotherapy, an overall increased preoperative risk of anastomotic leakage, or after anastomotic leakage.^8,9^ In our multivariable regression analyses of fecal incontinence and major LARS, we adjusted for most of these factors. Indeed, after statistical adjustments, fecal incontinence was no longer associated with a temporary ileostomy or colostomy.

Although a temporary ileostomy remained significantly associated with major LARS in multivariable regression, we found no association between a temporary colostomy and major LARS after the statistical adjustments. We postulate that this difference in functional outcomes between a previous ileostomy and a previous colostomy is related to the reasons for creating the 2 different types of stomas. A temporary ileostomy, for instance, is chosen especially in patients who underwent neoadjuvant radiotherapy, which renders them prone to postoperative LARS.^25^ Another reason for the association between a temporary ileostomy and major LARS may be a difference in colonic microflora. The colonic microflora is known to be different after the creation of a temporary ileostomy.^30^ Possibly, the colonic microflora remains different after stoma reversal, resulting in a more liquid stool consistency and/or persisting diversion colitis.^30–32^ This may eventually cause major LARS. Further prospective and longitudinal research is needed in which patients are randomly assigned to a temporary ileostomy. In this way, we may overcome the bias of studying the more fragile patients receiving a temporary stoma. This thought is supported by a small randomized controlled trial that showed no significant difference in LARS years after randomly creating a temporary stoma after rectal cancer surgery.^13^ Finally, the LARS score may not be detailed enough to capture patient’s postoperative bowel function, and it may be difficult to interpret because it contains a mixture of symptoms.^33,34^

Despite more fecal incontinence and major LARS in patients with an ileostomy and more major LARS after a colostomy, we did not find a worse quality of life in these patients. These results are corroborated by a previous study on quality of life after stoma reversal.^11^ It is known that stoma-related problems have a deteriorating effect on quality of life.^35^ Therefore, we postulate that patients are relieved by the stoma reversal and may cope well with the bowel function problems because these problems may have less influence on their daily lives than their previous stoma-related problems.

There is no consensus on the best timing of stoma reversal. We found a median time to stoma reversal of 4 months, although the range was very broad. In previous studies, time to stoma reversal varied between 2 weeks and 1 year, with most studies advising stoma reversal within 3 months.^16^ A recent systematic review and meta-analysis showed that ileostomy reversal within 6 months resulted in less major LARS, whereas reversal after 1 year resulted in more major LARS.^12^ An increasing number of studies investigated very early stoma reversal; one study even reported successful bowel continuity restoration only after 3 weeks, albeit in a small sample.^36^ Another meta-analysis reported comparable outcomes of ileostomy reversal within 2 weeks.^37^ A recent randomized controlled trial with a large sample investigated the difference between 2 and 12 weeks. However, the study was stopped early because of adverse feasibility and higher morbidity in the 2-week group.^14^ A Dutch study reported better functional outcomes and quality of life in patients who had their stomas reversed within 3 months, although the follow-up of this study was limited to 19 months.^16^ The current study does not show a relationship between time to stoma reversal and long-term functional outcomes or quality of life. Therefore, the best time to stoma reversal should be decided for each patient individually, especially in the case of postoperative complications.

The current study is limited by its cross-sectional design, which limited the comparison with preexisting bowel functioning. We also acknowledge that the statistical comparison of patient populations with and without a temporary stoma is limited by the clinical decision of the surgeon to construct a temporary stoma. Furthermore, the exclusion of patients who did not have their stomas reversed or who did not survive may have led to an overestimation of the functional outcomes after a temporary stoma. Moreover, the responders may theoretically have been in better physical condition than the nonresponders. Nevertheless, we had a response rate of 86%, and analysis between the responders and nonresponders showed no significant differences, except that the responders were slightly younger than the nonresponders. The large study group allowed us to correct for different associated factors in multivariable regression analysis.

## CONCLUSION

In the long term, patients with a temporary ileostomy after rectal cancer surgery experience more fecal incontinence and major LARS. Patients with a temporary colostomy experience more major LARS. Nevertheless, multivariable analysis showed that after adjustments for important reasons for creating a temporary stoma (eg, low anastomotic height, neoadjuvant radiotherapy, anastomotic leakage), fecal incontinence was neither associated with a temporary ileostomy nor with a temporary colostomy. Therefore, it seems that the reasons for creating the temporary stoma, rather than the stoma itself, lead to worse functional outcomes. Time to reversal of the temporary stoma does not seem to influence the long-term functional outcomes or quality of life.

## ACKNOWLEDGMENTS

The authors thank all patients for participating. They also acknowledge I.A.M. ten Vaarwerk and E. Visser for their help with processing the digital data and T. van Wulfften Palthe, PhD, for correcting the English article.

## Supplementary Material


