# Supplementary material for: The Effect of a Temporary Stoma on Long-term Functional Outcomes Following Surgery for Rectal Cancer
Source: Dis Colon Rectum. 2023 Dec 20;67(2):291–301. doi: 10.1097/DCR.0000000000003009 (PMC10769172; doi:10.1097/DCR.0000000000003009)
Supplement: Supplementary file 1 [file dcr-67-0291-s002.pdf]

Supplemental Digital Content 1. Temporary stomas in the responders and non-responders

	**Responders** No. (%)	**Non-responders** No. (%)	***p* value**
**Overall**	656 (100.0)	103 (100.0)	
Temporary stoma			
No	318 (48.5)	49 (48.0)	*0.932*
Ileostomy	208 (31.7)	34 (33.3)	
Colostomy	130 (19.8)	19 (18.6)	
