# Supplementary material for: The Effect of a Temporary Stoma on Long-term Functional Outcomes Following Surgery for Rectal Cancer
Source: Dis Colon Rectum. 2023 Dec 20;67(2):291–301. doi: 10.1097/DCR.0000000000003009 (PMC10769172; doi:10.1097/DCR.0000000000003009)
Supplement: Supplementary file 2 [file dcr-67-0291-s003.pdf]

Supplemental Digital Content 2. Reported reasons for constructing a temporary stoma

	**Ileostomy** No. (%)	**Colostomy** No. (%)
**Overall**	208 (100.0)	130 (100.0)
Bowel obstruction and needing neoadjuvant treatment	2 (1.0)	27 (20.8)
Bowel inflammation	0 (0.0)	1 (0.8)
According to preoperative plan	5 (2.4)	0 (0.0)
Neoadjuvant radiotherapy	34 (16.3)	20 (15.4)
Avoiding traction on colon	1 (0.5)	0 (0.0)
Comorbidity/condition/extensive surgical procedure	0 (0.0)	10 (7.7)
(Very) low anastomosis	25 (12.0)	24 (18.5)
Questionable condition anastomosis	5 (2.4)	18 (13.8)
Emergency setting	0 (0.0)	8 (6.2)
Anastomotic leakage	9 (4.3)	7 (5.4)
Not reported	127 (61.1)	15 (11.5)
