# Supplementary material for: The Effect of a Temporary Stoma on Long-term Functional Outcomes Following Surgery for Rectal Cancer
Source: Dis Colon Rectum. 2023 Dec 20;67(2):291–301. doi: 10.1097/DCR.0000000000003009 (PMC10769172; doi:10.1097/DCR.0000000000003009)
Supplement: Supplementary file 3 [file dcr-67-0291-s004.pdf]

Supplemental Digital Content 3. Correlations between time to stoma reversal and quality of life

	**Spearman correlation coefficient** rho	***p* value**
Physical functioning	-0.042	*0.454*
Role – physical	-0.027	*0.634*
Bodily pain	-0.120	*0.031* *
Mental health	-0.047	*0.406*
Role – emotional	-0.030	*0.597*
Social functioning	-0.063	*0.264*
Vitality	-0.120	*0.033* *
General health perceptions	-0.074	*0.187*

* Statistical significance of $p < 0.05$
